# Management of chronic lateral instability due to lateral collateral ligament deficiency after total knee arthroplasty: a case report

**DOI:** 10.1186/1752-1947-4-144

**Published:** 2010-05-21

**Authors:** Aasis Unnanuntana, James E Murphy, William J Petersilge

**Affiliations:** 1 Department of Orthopaedic Surgery, Siriraj Hospital, Mahidol University, 2 Prannok Road, Bangkok, Thailand; 2 Department of Orthopaedics, University Hospitals Case Medical Center, Case Western Reserve University School of Medicine, 11100 Euclid avenue, Cleveland, OH, USA

## Abstract

**Introduction:**

Lateral instability following total knee arthroplasty (TKA) is a rare condition with limited report of treatment options. The objective of this case presentation is to demonstrate the outcomes of different surgical procedures performed in a single patient with lateral collateral ligament (LCL) deficiency.

**Case presentation:**

We present a case of chronic lateral instability due to LCL deficiency after primary TKA in a 47-year-old Caucasian woman with an obesity problem. Multiple treatment options have been performed in order to manage this problem, including the following: ligament reconstruction; combined ligament reconstruction and constrained implant; and rotating-hinge knee prosthesis that was the most recent surgery. All ligament reconstruction procedures failed within one year. The varus-valgus constrained prosthesis provided stability for six years.

**Conclusions:**

Ligament reconstruction alone cannot provide enough stability for the treatment of chronic lateral instability in patients with obesity problems and LCL deficiency. When the reconstruction fails, a salvage procedure with rotating-hinge knee is still available.

## Introduction

Instability is one cause for aseptic failure following total knee arthroplasty (TKA). Varus-valgus instability can result from ligament imbalance, component malalignment, component loosening, bone loss, bone fracture, polyethylene wear, or collateral ligament failure. Medial (valgus) instability is much more common than lateral (varus) instability, and several repair techniques and treatment options are described in the literature [1-3].

To the best of our knowledge, however, no such reports exist for lateral instability resulting from lateral collateral ligament (LCL) deficiency after TKA. We present a case in which various surgical treatment options were performed to correct lateral instability. The objective of this case presentation is to demonstrate the outcomes of different surgical procedures performed in a single patient with LCL deficiency.

## Case presentation

A 47-year-old Caucasian woman presented in our institution 18 months after undergoing primary left TKA (Insall-Burstein II, Posterior-Substitute, Zimmer, Warsaw, IN). She had a post-operative history of recurrent instability and multiple episodes of knee dislocation. Her medical history was significant for severe psychiatric disorders, including bipolar disease and depression, and morbid obesity (body mass index (BMI) = 61 kg/m^2^). Her knee stability was tested under fluoroscopic guidance. The LCL appeared to be non-functional, as the knee fully opened to varus stress in both flexion and extension. Non-operative management with a knee immobilizer was prescribed.

Our patient returned two weeks later with another episode of knee dislocation. Closed reduction was achieved. A biceps femoris advancement was performed to treat the instability of the knee to varus stress. Stability was achieved for only seven months, as our patient began to feel lateral pulling with a resultant instability and, subsequently, further dislocations. Surgery was performed to reconstruct the ligament using an Achilles tendon allograft. The reconstruction, however, failed within 10 months.

A revision TKA using a more constrained implant (varus-valgus constrained implants) was then performed. Intra-operatively, all of the primary TKA components were well-fixed and in good position. However, the polyethylene tibial insert (Posterior Stabilized polyethylene insert, Zimmer, Warsaw, IN) demonstrated severe wear of the post. The previous ligamentous advancement and allograft reconstruction had completely avulsed from the tibia distally. No soft tissue existed to provide appropriate lateral support. A varus-valgus constrained polyethylene insert with a thickness of 17 mm (LCCK, Zimmer, Warsaw, IN) was used. Intra-operatively, the revised implant provided good stability throughout flexion and extension.

**Figure 1:**
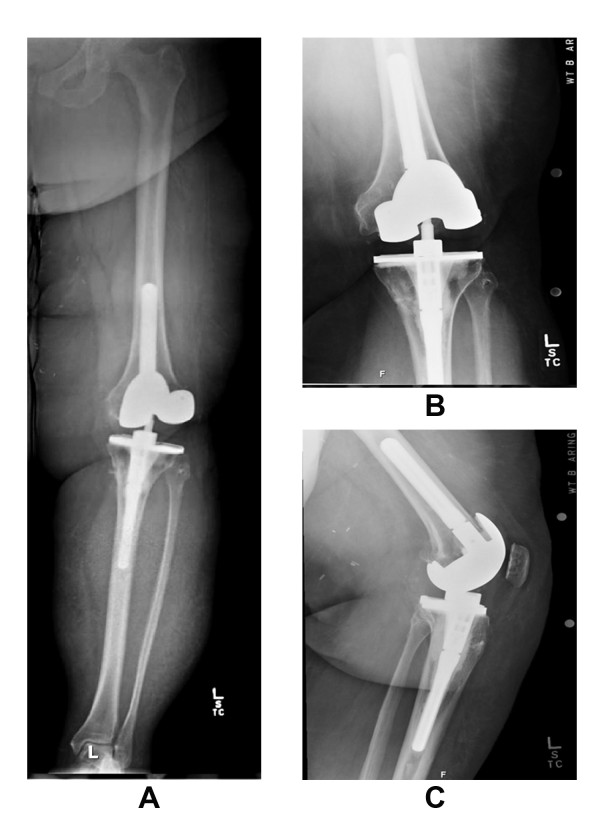
**Pre-operative radiographs long standing anteroposterior (A) anteroposterior (B) and lateral view (C) of the left knee of our patient, showing significantly increased gap on the lateral side**. There was no radiographic evidence of implant loosening. This lateral instability was secondary to the ligamentous failure.

Following revision surgery, the knee of our patient remained stable for six years without any clinical symptoms of instability. She then began noticing lateral knee pain and feelings of instability, but without frank dislocation. The pain worsened, and she fell twice after the onset of instability. On physical examination, she had lateral laxity with significant varus thrust. Five degrees of varus deformity existed as measured from the mechanical axis on a weight-bearing radiograph (Figure 1). Active and passive motion ranged from full extension to 110° flexion (further flexion was limited by impingement of the posterior soft tissues over her thigh and calf). Radiographic examination on our patient showed well-aligned prosthetic components with no evidence of implant loosening. The knee opened widely on the lateral as compared to the medial side (Figure 1). The knee society clinical rating and function scores were 22 and five points, respectively.

**Figure 2:**
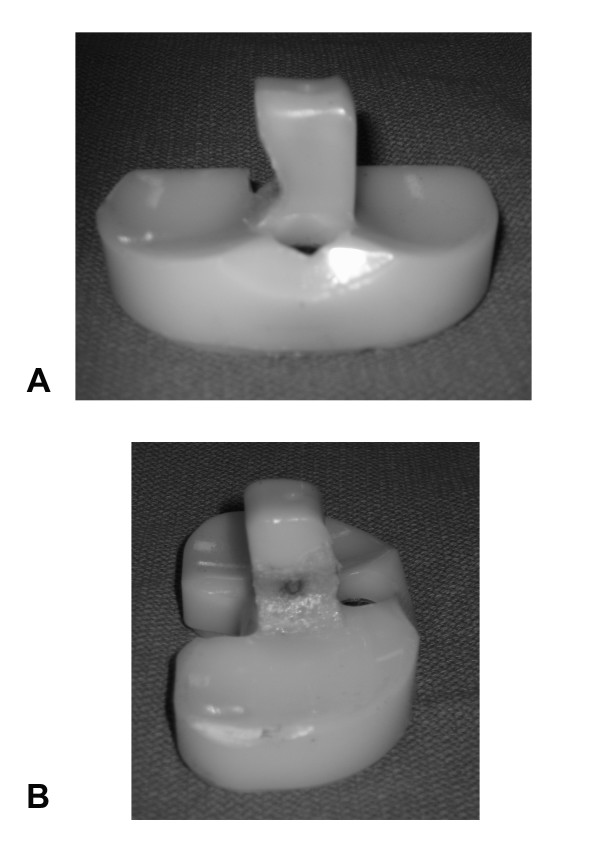
**Photograph showing significant wear along the medial side of the post of polyethylene insert**. **(A)**. The wear went through polyethylene exposing central metal post **(B)**.

All treatment options were discussed with our patient. She refused to undergo arthrodesis or any kind of hinged knee prosthesis, and instead requested a more conservative procedure. Due to her previous experience, which had performed well for approximately six years with only varus-valgus constrained polyethylene, we decided to perform a combination procedure of revision to a new polyethylene insert and an allograft reconstruction of the LCL with Achilles tendon and a calcaneal bone block. At surgery, the post of the LCCK polyethylene insert was grossly deformed along the medial side and severely worn through the polyethylene down to the central metal post (Figure 2). Post-operatively, our patient had persistent drainage. The post-operative culture grew *Staphylococcus aureus*. Our patient was taken back to surgery for irrigation and debridement with polyethylene exchange. Unfortunately, she failed to respond to the treatment, thus a two-stage revision surgery was performed. After six weeks of an antibiotic cement spacer combined with systemic antibiotics, the infection was cleared, shown by normal erythrocyte sedimentation rate (ESR), C-reactive protein (CRP) and negative culture from aspiration. At the second stage surgery, the frozen section revealed no evidence of acute inflammation, and her knee was revised to rotating-hinge prosthesis.

Post-operatively, full range of motion and full weight bearing were allowed. The wound healed well without any complication. Our patient has no evidence of infection 18 months after surgery. The knee is stable with active flexion to 100° and a 25° extension lag. The knee society clinical rating and function scores are 65 and 60 points, respectively. Radiographs showed well-aligned prosthetic components without evidence of implant failure.

## Discussion

Lateral instability is one of the causes of failure after TKA. There is very little information in the literature documenting the incidence of this fortunately rare condition and even less information discussing the treatment options and results. In general, instability following TKA can be managed by different interventions depending on severity of the instability and the condition of the collateral ligaments. Treatments include bracing, isolated ligament advancement or reconstruction alone, ligament reconstruction in conjunction with constrained TKA devices (varus-valgus constrained implants), hinged knee implants, and arthrodesis.

LCL reconstruction has been described in the literature [4,5], although these procedures have usually been performed in trauma patients. Pritsch *et al*. concluded that ligament reconstruction alone could not be expected to stabilize the unstable knee replacement based on a series of seven surgeries for medial instability, all of which failed [3]. Similarly, the ligament reconstruction procedures in our patient failed within one year. Vince *et al*. emphasized the importance of correcting factors such as malalignment from adjacent joint pathology or extra-articular deformity and neuromuscular pathology [6]. In addition, previous studies have shown that the higher BMI of our patient at the time of ligament reconstruction is predictive of poor outcome [7]. Therefore, because of her obesity, the probability of failure with any reconstructive procedures for our patient was high.

Varus-valgus knee stability is derived from transfer of the joint contact load between the condyles, muscle forces, the collateral ligaments, and, in the case of TKA, mechanical constraints provided between the implant components. Generally, rotating-hinge knee implants (linked constrained prostheses) are indicated when the collateral ligaments are absent or beyond reconstruction [8]; however, no data exist to justify whether less constrained implants (unlinked constrained prosthesis) are inadequate in this situation. Increasing component constraint can also increase forces transmitted to the implant fixation interfaces, which may lead to premature aseptic loosening. Therefore, in our opinion, the use of less constrained devices with ligament reconstruction is more conservative than rotating-hinge knee implant, especially for young, active patients.

This opinion is supported by our patient, who did not develop symptoms of instability for about six years after revision to only varus-valgus constrained polyethylene insert. We believe that a combined surgical procedure of exchange to a new constrained polyethylene insert and ligament reconstruction allows initial coronal stability from the implant while the reconstructed graft incorporates, eventually providing additional long-term stability. Unfortunately, no evidence-based studies exist to support this concept.

Total joint arthroplasty has the risk of infection. The infection rate of ligament reconstruction in the native knee is low [9], but we know of no study reporting the infection incidence following collateral ligament reconstruction in TKA. Our patient had an acute post-operative infection that lead to debridement and removal of both the prosthesis and the allograft. Therefore, such a combined procedure should be limited to patients who carry low risk of infection.

The main limitation of our case is that the follow-up of the most recent procedure, rotating-hinge TKA, is short (18 months). However, our objective was to demonstrate the outcome of different surgical procedures performed to treat this complex situation. Pour *et al*. reported that the survival rate of rotating-hinge TKA was 79.6% at one year and 68.2% at five years with revision or re-operation as the end point [10]. Although such studies demonstrate that the complication rate of rotating-hinge TKA is high [10,11], the rotating-hinge device remains a viable option in the face of failed multiple previous surgical procedures such as in our patient.

## Conclusions

Ligament reconstruction alone cannot provide enough stability for the treatment of chronic lateral instability in obese patients with LCL deficiency. As a general rule, it is recommended that the minimum amount of constraint necessary to achieve stability should be used. The varus-valgus constrained prosthesis may provide short to intermediate stability of the knee. Although the concept of combined procedure with ligament reconstruction and using varus-valgus constrained implant is somewhat interesting, the risk of infection is high. Should the reconstruction fail, a salvage procedure with rotating-hinge knee devices is still available.

## Consent

Written informed consent was obtained from our patient for publication of this case report and any accompanying images. A copy of the written consent is available for review by the Editor-in-Chief of this journal.

## Competing interests

The authors declare that they have no competing interests.

## Authors' contributions

AU was the principal investigator of the study, conducted the collection of data and was involved in drafting the article. JEM was involved in drafting the article. WJP helped in manuscript preparation and operated upon our patient. All authors read and approved the final manuscript.
